# Growth Suppression of a Robust Bacterium *Methylobacterium extorquens* by Porous Materials with Oxygen Functional Groups

**DOI:** 10.3390/life13112185

**Published:** 2023-11-09

**Authors:** Takeshi Mori, Yuta Ogawa, Izuki Endo, Keiichiro Matsushima, Jun Noda

**Affiliations:** 1 Industrial Research Institute, Hokkaido Research Organization, Sapporo 060-0819, Japan; 2 School of Veterinary Medicine, Rakuno Gakuen University, Ebetsu 069-8501, Japan; jnoda@rakuno.ac.jp (J.N.); 3 Department of Applied Chemistry and Bioscience, Chitose Institute of Science and Technology, Chitose 066-8655, Japan; 4 School of Human Science and Environment, University of Hyogo, 1-1-12 Shinzaike-Honcho, Himeji 670-0092, Japan; izok@shse.u-hyogo.ac.jp

**Keywords:** methylobacterium, porous materials, anti-bacterial materials, oxygen functional groups

## Abstract

Suppressing the growth of *Methylobacterium* species without the use of toxic chemicals has been a challenging task owing to their robustness against previous antimicrobial techniques. In this work, we prepared porous materials with various numbers and types of oxygen functional groups and investigated their ability to suppress the growth of *Methylobacterium extorquens*. It turned out that the number and type of oxygen functional groups in the porous materials greatly affected the growth of the bacterium. Three porous materials (resorcinol–formaldehyde gel (RF), hydrothermally treated RF (RFH), and Wakkanai siliceous shale (WS)) were tested, and RF exhibited the best performance in suppressing the growth of the bacterium. This performance is possibly due to abundant phenolic groups in the porous material.

## 1. Introduction

*Methylobacterium* species are Gram-negative bacteria characterized by their distinct pink-pigmented colony and unique ability to grow on C1 compounds such as methanol and methylamine [1]. This feature enables the species to inhabit plant leaf surfaces; the species can produce plant growth hormones (e.g., cytokinin and indole-3-acetic acid) from a trace amount of gaseous C1 compounds such as methanol released from plants [2,3]. Owing to this feature, the *Methylobacterium* species are promising candidates for substitutes of agrochemicals [4], whose residues often cause severe contamination in arable lands. The *Methylobacterium* species do not show high toxicity; however, the species are known to cause some opportunistic infections, which are illnesses that occur in people with weakened immune systems [5,6]. A clinical report showed that the *Methylobacterium* species were detected in biopsy specimens from patients (e.g., ulcers, phlegm, and pleural effusion) [6]. The entry of the *Methylobacterium* species into such people can lead to the occurrence of severe infections such as pneumonia, catheter-related blood stream infections, and bacteremia [6]. *Methylobacterium mesophilicum*, *Methylobacterium zatmanii,* and *Methylobacterium extorquens* are the three most commonly reported species isolated from biopsy specimens [7]. The species can be ubiquitously found in nature (e.g., soil, plants, and watersheds) [8]; therefore, techniques to suppress the growth of the *Methylobacterium* species are desired to prevent the opportunistic infections caused by the species.

While the *Methylobacterium* species possess such potential toxicity, the species are chemically robust and their growth can hardly be suppressed by typical antimicrobial techniques [9]. *Methylobacterium* species can use short-chain alcohols as a carbon source. *Methylobacterium* species contain enzymes that can assimilate one and multi-carbon substrates including short-chain alcohols [1,10]; therefore, alcohol disinfectant is not an effective method to suppress their growth. *Methylobacterium* species survive even under exposure to ultraviolet light-emitting diodes [11,12]. A study showed that *Methylobacterium* species contain a chemical compound similar to avobenzone, which is known to absorb ultraviolet light [13]. This feature could be one of the major factors responsible for the ultraviolet light resistance of the species. To suppress such robust *Methylobacterium* species, Yano et.al. proposed the use of a combination of alcohols and surfactants [14,15]. It turned out that a combination of phenolic compounds (e.g., phenethyl alcohol and phenylglycol) with benzalkonium chloride effectively suppressed the growth of *Methylobacterium* species. Phenolic compounds played a role as a “penetration enhancer” to facilitate the access of benzalkonium chloride to the bacteria. Since the use of benzalkonium chloride alone does not suppress the growth of *Methylobacterium* species, the use of phenolic compounds is crucial for this suppression. Such compounds are typically provided in liquid form and are used by spraying or pouring them onto the bacteria. Such liquid products were developed for cleaning purposes for sinks and bathtubs. Meanwhile, there are also huge demands on suppressing the growth of *Methylobacterium* species in waste-water management and air-purification in hospitals. For such purposes, the use of such chemicals is impractical because of their high toxicity and low cost-efficiency.

Resorcinol–formaldehyde (RF) gels are solid polymers synthesized via the polycondensation of resorcinol with formaldehyde [16]. RF gels have phenolic hydroxyl groups derived from the precursor resorcinol. It is expected that RF gels can suppress the growth of *Methylobacterium* species owing to their molecular structure, which is analogous with the phenolic compounds. These RF gels and their carbonized materials are essentially porous, and their surface areas are often as high as and/or more than several hundreds of square meters per gram. Owing to this feature, the materials are often used as adsorbents [17], catalyst supports [18], and electrode materials [19,20]. The use of such polymers is more environmentally friendly than conventional spraying methods because it does not generate liquid waste containing hazardous chemicals. In this work, we designed RF gels that can suppress the growth of one *Methylobacterium* species, *Methylobacterium extorquens* (*M. extorquens*). Through culture tests of *M.extorquens* with various solid polymers and minerals, we elucidate the material properties required for the suppression of the growth of the bacterium.

## 2. Materials and Methods

### 2.1. Materials

Resorcinol (98%), formalin (aqueous solution of formaldehyde at a concentration of 37%), sodium carbonate (99.8%), diammonium hydrogen phosphate (NH_4_)_2_HPO_4_, 99%), potassium chloride (KCl, 99.5%), magnesium sulfate heptahydrate (MgSO_4_ 7H_2_O, 99.5%), and glycerol (99%) were purchased from Kishida chemical Co., Ltd. (Osaka, Japan). Agar powder was purchased from Hayashi Pure Chemical Ind. Co., Ltd. (Osaka, Japan). Yeast extract (yeast extract granulated, Millipore) was purchased from Sigma Aldrich (Burlington, WA, USA). Peptone was purchased from Kyokuto pharmaceutical industrial Co., Ltd. (Tokyo, Japan). A type strain of *M. extorquens*, which is derived from ATCC BAA-2500, was purchased from Microbiologics (Saint Cloud, FL, USA). Wakkanai siliceous shale (WS) was purchased from Wakkanai greenfactory Co., Ltd. (Wakkanai, Japan). WS was used in the culture test as received.

### 2.2. Synthesis of Resorcinol–Formaldehyde Gel (RF) and Hydrothermally Treated RF Gel (RFH)

In a typical manner, resorcinol (25 g), formalin (36.9 g), and sodium carbonate (24.1 mg) were added to distilled water (29.7 g) and stirred until they dissolved. The mixture was poured into a polypropylene (PP) container which was sealed with a cap. The container was placed in a chamber and kept at 60 °C for 72 h to turn the mixture into hydrogel. The obtained hydrogel was removed from the container and dried at 120 °C for 48 h to obtain resorcinol–formaldehyde gel (RF). The hydrothermally treated RF gel (RFH) was prepared from the same mixture of resorcinol, formalin, sodium carbonate, and distilled water. This mixture (30 g) was poured into a Teflon container with a cap. This container was placed in a steel jacket and tightly sealed. This jacket was placed in a chamber kept at 250 °C for 24 h. The content in the Teflon container was collected and then dried in a chamber kept at 120 °C for 48 h.

### 2.3. Characterization of the Porous Materials (RF, RFH, and WS)

An X-ray fluorescence spectrometer (XRF, ZSX Primus II, Rigaku Corporation (Tokyo, Japan)) was used for elemental analysis of the porous materials (RF, RFH, and WS). A field emission scanning electron microscope (FE-SEM, JSM-7001F, JEOL (Tokyo, Japan)) was used to observe the morphologies of the porous materials. The samples were coated with platinum prior to observation.

The textual pore properties of the porous materials were characterized by nitrogen adsorption experiments at 77 K. A portion of the porous materials (approximately 0.1 g) was placed in a glass-made sample tube. This tube was attached to an adsorption apparatus (BELSORP MAX, Microtrac BEL (Osaka, Japan)) to measure the specific surface area, *S*_BET_ [m^2^ g_solid_^−1^], of the porous materials. This *S*_BET_ was calculated by using the Brunauer–Emmett–Teller (BET) method [21]. The details of this calculation are shown in the Supporting Information. Prior to the adsorption experiments, the samples were dried at 120 °C for 6 h under vacuum conditions (<0.1 kPa).

The number of phenolic groups in the porous material was indirectly quantified by a water adsorption experiment (25 °C) using the same apparatus. The amount of water adsorbed at *p/p*_0_ = 0.15, *V*_H2O,0.15_ [cm_H2O_^3^ g_solid_^−1^], relates to the monolayer coverage of water molecules on the solid surface. The amount of adsorbed water per unit surface area, *Γ* [nm^−2^], can be calculated by dividing *V*_H2O,0.15_ by the specific surface area of the solid *S*_BET_ [m^2^ g_solid_^−1^] (Equation (1)). *N*_A_ and *V*_molar_ represent the Avogadro’s number (6.023 × 10^23^ mol^−1^) and the molar volume of nitrogen gas (22,414 cm^3^ mol^−1^), respectively. This *Γ* [nm^−2^] was used as an index representing the number of the phenolic groups that the porous material possesses. The surface functional groups of the porous materials were also characterized by using a Fourier-transform infrared (FTIR) spectrometer (Spectrum 100, Perkin Elmer (Shelton, WA, USA)).

(1) $$\Gamma =\frac{V_{H2O,0.15}}{{\;S}_{\mathrm{BET}\;}}\times \frac{N_{A}}{V_{\mathrm{molar}}}\times 10^{-18}$$

### 2.4. Microbiological Culture Tests of M. extorquens

#### 2.4.1. Preparation of Methanol-Mediated Salts (MMS) Agar Medium

The methanol-mediated salts (MMS) agar medium was prepared typically by mixing 3.0 of diammonium hydrogenphosphate, 0.5 g of potassium chloride, 0.05 g of magnesium sulfate heptahydrate, and 0.25 g of yeast extract in 500 mL of distilled water until the mixture turned into clear solution. Then, 10 g of agar was added to the solution, and the mixture was autoclaved at 121 °C for 20 min. The solution was cooled down to approximately 70 °C and then 5.0 mL of methanol was added. The mixture was poured into a disposable Petri dish made of polystyrene and cooled down to ambient temperature to induce gelation of the mixture.

#### 2.4.2. Preparation of Stock Solution of *M. extorquens*

A type strain of *M. extorqens* was streaked on the MMS agar medium, and it was placed in an incubator kept at 30 °C for 7 days. Pink colonies of *M. extorqens* were formed on the medium. Several pieces of the pink colonies were taken by an inoculation loop and put in an aliquot of the liquid MMS medium, which is a liquid medium prepared by the recipe for the MMS agar medium without adding agar. This stock solution (titer: 1.3 × 10^9^ CFU mL^−1^) was agitated by a vortex mixer and then stored in the incubator kept at −30 °C.

#### 2.4.3. Culture Tests of *M. extorquens* with the Porous Materials

A porous material (1.0 g, either RF or RFH) and an aliquot of the stock solution of *M. extorqens* (0.1 mL) were added to the glycerol–peptone (GP) liquid medium (50 g) in a 300 mL Erlenmeyer flask. The GP liquid medium was prepared typically by mixing 2.5 g of peptone and 2.5 g of glycerol in 250 mL of distilled water. The flask was horizontally shaken by a shaking incubator with a speed of 150 rpm at 30 °C for 7 days. After this culturing process, a portion of the mixed solution (0.1 mL) in the flask was transferred to an Eppendorf tube with the liquid MMS medium. This solution was diluted with MMS medium (dilution rate: 10^2^–10^7^) for colony counting tests. A portion of this diluted solution was streaked on the MMS agar medium and cultured at 30 °C for 7 days in a static incubator. The number of pink colonies on the medium, *n*, was counted to calculate the titer in the mixed solution (titer [CFU mL^−1^] = *n* [CFU] × dilution ratio (10^2^–10^7^) [-]/0.1 [mL]). A culture test without the addition of any porous material was also performed for the control experiment. The culture test with WS was also conducted to investigate the effect of oxygen functional groups on the growth suppression of the bacteria by comparing its result against that of RF. Both WS and RF possess similar a number but different types of oxygen functional groups.

## 3. Results

### 3.1. Characterization of the Porous Materials

#### 3.1.1. Elemental Analysis

Table 1 shows the elemental analysis of the porous materials (RF, RFH, and WS) determined by XRF. The major components of RF were oxygen (38.3%) and carbon (61.6%). The content of oxygen in RFH (32.4%) was approximately 6% lower than that of RF. The content of carbon in RFH (67.6%) was approximately 6% higher than that of RF. This result indicates that a part of the oxygen functional groups was decomposed by the hydrothermal treatment at 250 °C. The major components of WS were oxygen (55.2%) and silicon (34.4%). 

#### 3.1.2. Morphology

**Figure 1:**
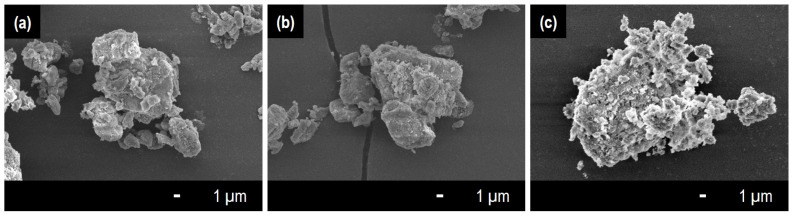
FE-SEM images of the porous materials used in the culture tests: (**a**) RF, (**b**) RFH, and (**c**) WS.

The morphologies of the porous materials were observed by FE-SEM (Figure 1a–c). The sizes of the particles of all porous materials were in the range of several to several tens of micrometers. The micrographs show that no pores were observed on the surface of the particles. Considering that *M. extorqens* is a rod-shaped microbe with a size of 0.8–1.2 µm × 1.0–8.0 µm [1], the porous materials do not possess pores and voids that can accommodate the bacteria.

#### 3.1.3. Textual Properties

The textual properties of the porous materials are summarized in Table 2. The specific surface areas, *S*_BET_, of RF and RFH were almost the same value (RF: 240 m^2^g^−1^ and RFH: 260 m^2^g^−1^), while that of WS (WS: 120 m^2^g^−1^) was lower than that of RF and RFH. Further analysis of their textual properties was conducted by analyzing the shape of the nitrogen adsorption–desorption isotherms measured at 77 K (Figure S1). The isotherm of RF was a type IV isotherm, which has large N_2_ uptake *V*_a_ at low and high relative pressure (low: *p*/*p*_0_ < 0.2 and high: *p*/*p*_0_ > 0.8) and whose *V*_a_ converges to a certain value when *p*/*p*_0_ approaches unity. This suggests that RF possesses two classes of pores: one of them is a micropore (the pore width is less than 2 nm) and another one is a mesopore (the pore width is in the range of 2–50 nm) [22]. RFH and WS showed similar isotherms; however, *V*_a_ does not converge to a certain value when *p*/*p*_0_ approaches unity. This is a type II isotherm, which indicates that RFH and WS possess a larger class of pore, a macropore (the pore width is larger than 50 nm), in addition to micropores [22].

#### 3.1.4. Oxygen Functional Group

The number of oxygen functional groups was indirectly quantified by the water adsorption experiments at 25 °C (Figure S2). The results are also summarized in Table 2. The number of oxygen functional groups was quantified by water adsorption uptakes at *p*/*p*_0_ = 0.15. This value of RFH was 30 cm^3^(STP) g^−1^, which was lower than that of RF (50 cm^3^(STP) g^−1^). This decrease in water adsorption uptake can be attributed to the decomposition of the hydrophilic oxygen functional groups such as phenolic groups. This result is consistent with the results of the elemental analysis by XRF. The uptake of WS at *p*/*p*_0_ = 0.15 was 29 cm^3^(STP) g^−1^. The apparent water adsorption uptake per unit surface area *Γ* [nm^−2^], which represents the number of oxygen functional groups, can be calculated by normalizing the water adsorption uptake at *p*/*p*_0_ = 0.15 by *S*_BET_. The *Γ* of RF and WS was 5.2 nm^−2^ and 6.2 nm^−2^, showing that those two porous materials possess similar hydrophilicity. Meanwhile, the *Γ* of RFH was 3.2 nm^−2^, which is approximately 60% of that of RF. This means that RFH is less hydrophilic than RF and WS.

### 3.2. Culture Tests of M. extorquens

#### 3.2.1. Culturing *M. extorquens* without the Porous Materials (Control Experiment)

**Figure 2:**
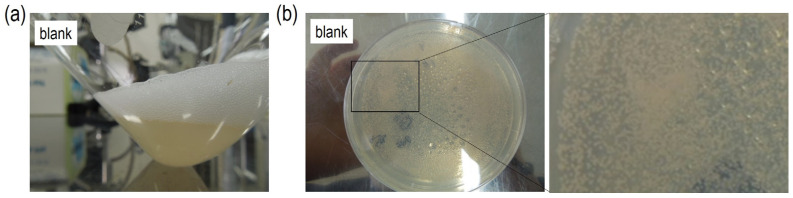
(**a**) A photograph of the cultured solution after culturing *M. extorquens* in GP media for 7 days; (**b**) photographs of an MMS agar plate streaked with the cultured solution of *M. extorquens* and incubated at 30 °C for 7 days.

Figure 2a shows a picture of the cultured solution after culturing *M. extorquens* in GP media for 7 days. It was a yellowish turbid solution, and bubbles were observed at the gas–liquid interface, which were presumably generated by the respiration of *M. extorquens.* Figure 2b shows a picture of an MMS agar plate, which was streaked with the cultured solution of *M. extorquens* and incubated at 30 °C for 7 days. Pink-pigmented colonies with a size of several millimeters were observed on the MMS agar plate. These features indicate that *M. extorquens* can be cultured in GP media and selectively grown on the MMS agar plate. The colony-forming unit of *M. extorquens* in the cultured solution was 1.6 × 10^8^ [CFU mL^−1^].

#### 3.2.2. Culturing *M. extorquens* with the Porous Materials

**Figure 3:**
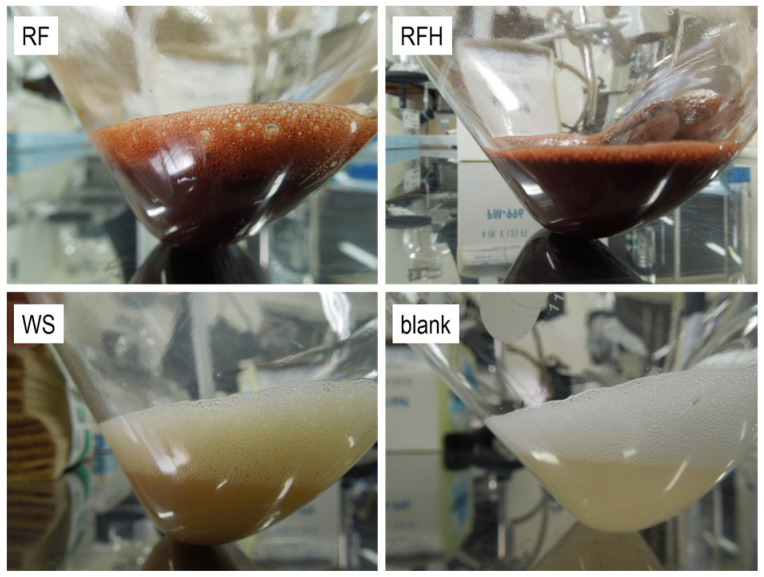
Photographs of the cultured solutions after culturing *M. extorquens* in GP liquid medium for 7 days with the porous materials.

*M.extorquens* was cultured in the presence of the porous materials, RF, RFH, and WS. These culture tests were conducted in GP liquid medium, which is favorable for the growth of *M. extorquens*. Figure 3 shows photographs of the solutions after culturing *M. extorquens* in the presence of one of the porous materials. The color of the cultured solution was dark red in the presence of RF and RFH, while it was brown in the presence of WS. 

**Figure 4:**
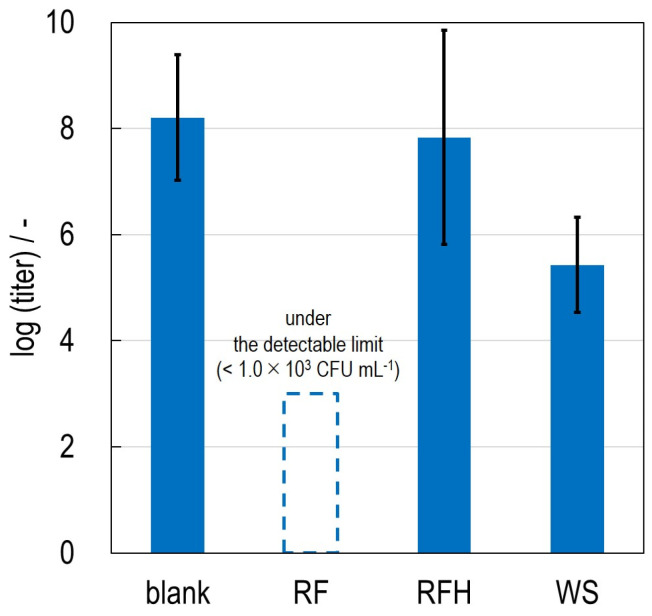
The results of the colony counting tests performed on the MMS agar plates in the presence (referred to as RF, RFH, and WS) and absence of the porous materials (referred to as blank). The colony counting tests were repeated three times (*n* = 3).

The results of the colony counting tests are shown in Figure 4. The colony-forming unit of *M. extorquens* in the cultured solution without any porous materials was 1.6 × 10^8^ [CFU mL^−1^]. On the other hand, that of the cultured solution in the presence of RF was less than the detectable limit (<1.0 × 10^3^ [CFU mL^−1^]). This means that RF decreases the number of *M. extorquens* in GP liquid medium. When the other types of porous materials (RFH and WS) were added to the cultured solution, the colony-forming units were 6.8 × 10^7^ [CFU mL^−1^] and 2.7 × 10^5^ [CFU mL^−1^], respectively. The colony-forming unit tends to decrease when the porous materials are present in the cultured solution.

## 4. Discussion

Regarding Section 3.2.2, it turns out that the colony-forming units of *M. extorquens* in the cultured solution depends on the type of porous material. The addition of porous materials decreased the colony-forming units. One of the common factors of these porous materials is possessing hydroxyl groups on their surfaces (FTIR spectrum shown in Figure S3; broad bands can be observed at approximately 3500 cm^−1^, which represents the stretching band of the hydroxyl group). Considering that the major components of RF and RFH are carbon and oxygen as shown in Table 1 and that both materials are derived from the same molecule (resorcinol: 1,3-dihydroxybenzene), the hydroxyl groups of RF and RFH are presumably phenolic groups. Meanwhile, the hydroxyl groups of WS appear to be silanol groups (Si-OH) considering that WS is mainly composed of silicone and oxygen. 

The relationships between the properties of the oxygen functional groups and the colony-forming units are summarized in Table 3. Since both RF and RFH possess phenolic groups but the number of functional groups is significantly different (RF: 5.2 nm^−2^; RFH: 3.2 nm^−2^), the effect of the number of functional group can be discussed by comparing RF and RFH. The colony-forming unit of the cultured solution with RF was less than the detectable value (<1.0 × 10^3^ [CFU mL^−1^]), while that with RFH was significantly higher (<6.8 × 10^7^ [CFU mL^−1^]). From this result, the growth of *M. extorquens* is suppressed more effectively in the presence of the porous materials with a higher number of phenolic groups. 

The effect of the type of oxygen functional group is also discussed by comparing the results of the culture tests using RF and WS. Both porous materials possess a similar number of functional groups (RF: 5.2 nm^−2^, WS: 6.2 nm^−2^), while the type of functional group is different (RF: phenolic group; WS: silanol group). The colony-forming unit of the cultured solution with WS was 2.7 × 10^5^ [CFU mL^−1^], which was significantly higher than that of RF (<1.0 × 10^3^ [CFU mL^−1^]). This suggests that the phenolic group can more efficiently suppress the growth of *M. extorquens* than the silanol group. It is known that such phenolic hydroxyl groups exhibit anti-fungal activity [23]. This work has shown that organosolv lignin from Japanese cedar tree suppressed the growth of wood-decay fungus (*Trametes versicolor*) due to its phenolic hydroxyl groups.

Since the *Methylobacterium* species can trigger some serious illnesses in immunocompromised patients, this RF gel technique can control and minimize the growth of the species. The bacteria can be spread in various forms (e.g., air containing aerosolized bacteria and wastewater containing the bacteria). RF gels can readily be molded into pellets and monoliths with a honeycomb-like structure [19,24], which enables one to continuously and efficiently inactivate the species. This technique can contribute to reducing health risks, which may be brought about by cutting-edge sustainable technology such as its application in biostimulants.

## 5. Conclusions

*Methylobacterium* species are robust bacteria due to their resistance to existing anti-microbial techniques; therefore, it is challenging to suppress the growth of *Methylobacterium* species. In this work, we developed a method to suppress the growth of *M. extorquens* by using porous materials with hydroxyl groups. The three types of porous materials, resorcinol–formaldehyde gel (RF), hydrothermally treated RF (RFH), and Wakkanai siliceous shale (WS), were prepared and used as porous materials to assess their ability to suppress the growth of *M. extorquens*. RF and WS exhibited the ability to suppress the growth of *M. extorquens* due to their oxygen functional groups. It turned out that the type and number of functional groups significantly affected the growth of the bacteria. This study showed that RF gel exhibited the best performance. Compared to the typical anti-microbial technique of spraying toxic chemicals to contaminate the environment, this method does not have such issues with toxic substances; thus, it is more environmentally friendly. 

## Figures and Tables

**Table 1:** Elemental analysis of the porous materials (RF, RFH, and WS) by XRF measurement.

Element	Percentage in Weight/%		
	RF	RFH	WS
oxygen	38.3	32.4	55.2
silicone	0	0	34.4
carbon	61.6	67.6	3.2
other elements	0.1	0	7.2 ^1^

^1^ The other elements in WS were aluminum (3.5%), iron (3.2%), potassium (0.8%), magnesium (0.4%), sodium (0.3%), calcium (0.2%), and titanium (0.1%).

**Table 2:** Textual pore properties of the porous materials (RF, RFH and WS).

Sample ID	*S*_BET_/m^2^ g^−1 a^	*V*_H2O,0.15_/cm^3^(STP) g^−1 b^	*Γ*/nm^−2 c^
RF	240	50	5.2
RFH	260	30	3.2
WS	120	29	6.2

^a^ BET surface area, *S*_BET_, was calculated by applying the BET method to the nitrogen adsorption isotherms at *p*/*p*_0_ = 0.05–0.2. ^b^ Water adsorption uptake, *V*_H2O,0.15_, was calculated by using the water adsorption isotherms at *p*/*p*_0_ = 0.15. ^c^ Apparent water adsorption uptake per unit surface area, *Γ*, was calculated by Equation (1) (*Γ* = *V*_H2O,0.15_ × *N*_A_ × 10^−18^/(*S*_BET_ × *V*_molar_)).

**Table 3:** Type and number of oxygen functional groups in the porous materials.

Type of Porous Material	Properties of the Oxygen Functional Group ^a^	Colony-Forming Units in the Cultured Solution/CFU mL^−1 b^
RF	phenolic group (*Γ =* 5.2 nm^−2^)	<1.0 × 10^3^
RFH	phenolic group (*Γ =* 3.2 nm^−2^)	6.8 × 10^7^
WS	silanol group (*Γ =* 6.2 nm^−2^)	2.7 × 10^5^
Blank	-	1.6 × 10^8^

^a^ Apparent water adsorption uptake per unit surface area, *Γ*, was calculated by Equation (1). This value corresponds to the number of oxygen functional groups. ^b^ Culture tests on MMS agar plates were conducted to measure the colony counting units (dilution rate: 10^5^).

## Data Availability

No new data were created or analyzed in this study. Data sharing is not applicable to this article.

## Supplementary Materials

The following supporting information can be downloaded at: https://www.mdpi.com/article/10.3390/life13112185/s1. Figure S1: Nitrogen adsorption and desorption isotherms of (a) RF, (b) RFH, and (c) WS measured at 77 K. Solid and open symbols indicate adsorption and desorption branches; Figure S2: Water adsorption and desorption isotherms of (a) RF, (b) RFH, and (c) WS measured at 298 K. Solid and open symbols indicate adsorption and desorption branches; Figure S3: Fourier-transform infrared spectrum: (red) RF, (blue) RFH, and (green) WS.
