# Supplementary material for: Growth Suppression of a Robust Bacterium Methylobacterium extorquens by Porous Materials with Oxygen Functional Groups
Source: Life (Basel). 2023 Nov 9;13(11):2185. doi: 10.3390/life13112185 (PMC10672207; doi:10.3390/life13112185)
Supplement: Supplementary file 1 [file life-13-02185-s001.zip › life-2664461-supplementary.pdf]

Supporting Information

The details of calculating the specific surface area from the nitrogen adsorption experiment

Nitrogen adsorption isotherm $V_a = f\,(p\ /\ p_0)$, which represents the amount of adsorbed nitrogen molecules on the solid surfaces, V_a [$cm_{N2}^3\ g_{solid}^{-1}$], as a function of relative pressure of nitrogen gas, $p\ /\ p_0$ [-] (p [kPa] represents the pressure of nitrogen gas in the sample tube and p_0 [kPa] represents the vapor pressure of nitrogen gas at 77 K (101.3 kPa)). The monolayer coverage, V_m, which is the amount of nitrogen molecules to cover the surface of the solid, can be calculated by fitting the nitrogen adsorption isotherm with the BET equation (equation S1, the value c relates to the adsorption heat and can also be obtained by the fitting). Specific surface area of the solid S_{BET} [$m^2\ g_{solid}^{-1}$] can be calculated by using V_m and equation S2, where V_{molar} represents the molar volume of nitrogen gas (22414 $cm^3\ mol^{-1}$), σ represents the cross-sectional area of the molecule of nitrogen gas (0.162 nm^2) and N_A represents the Avogadro's number ($6.023\times10^{23}\ mol^{-1}$).

$$\frac{p}{V_a\,(p_0 - p)} = \frac{1}{V_m\ C} + \frac{C - 1}{V_m\,C} \times \frac{p}{p_0} \qquad \text{(S1)}$$

$$S_{BET} = \frac{V_m}{V_{molar}} \times N_A \times \sigma \qquad \text{(S2)}$$

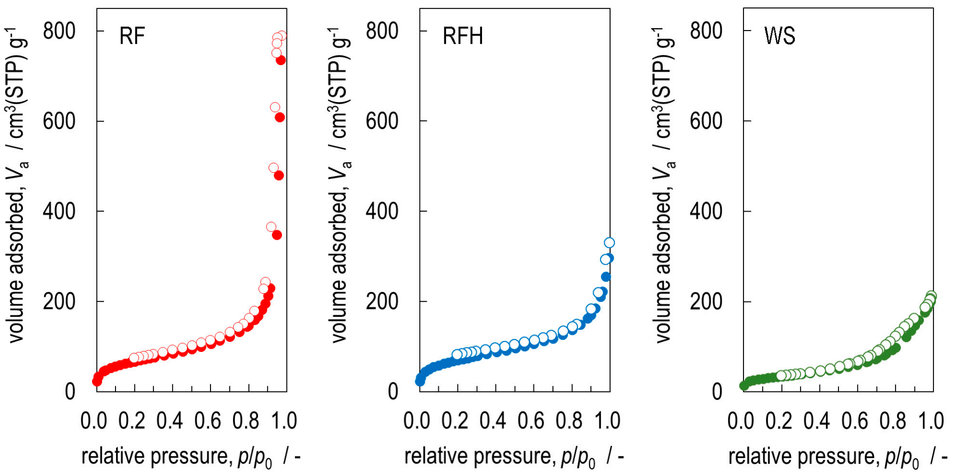

Figure S1. Nitrogen adsorption and desorption isotherms of RF, RFH and WS measured at 77 K. Solid and open symbols indicate adsorption and desorption branches, respectively

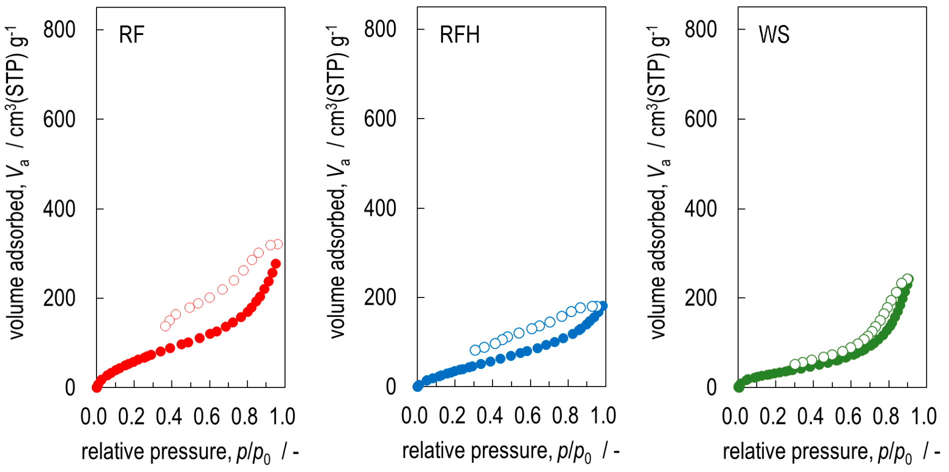

Figure S2. Water adsorption and desorption isotherms of RF, RFH and WS measured at 298 K. Solid and open symbols indicate adsorption and desorption branches, respectively.

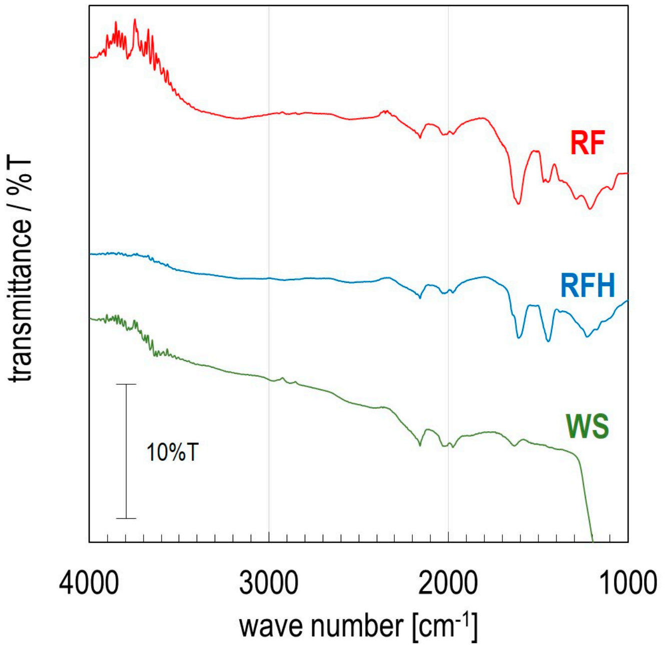

Figure S3. Fourier transform infrared spectrum: (red) RF, (blue) RFH and (green) WS.
